# Heat Stress Effects on Animal Health and Performance in Monogastric Livestock: Physiological Responses, Molecular Mechanisms, and Management Interventions

**DOI:** 10.3390/vetsci12050429

**Published:** 2025-04-30

**Authors:** José A. M. Prates

**Affiliations:** 1 CIISA—Centro de Investigação Interdisciplinar em Sanidade Animal, Faculdade de Medicina Veterinária, Universidade de Lisboa, Av. da Universidade Técnica, 1300-477 Lisboa, Portugal; japrates@fmv.ulisboa.pt; 2 Associate Laboratory for Animal and Veterinary Sciences (AL4AnimalS), Av. da Universidade Técnica, 1300-477 Lisboa, Portugal

**Keywords:** heat stress, monogastric livestock, oxidative stress, nutritional interventions, immune function, thermoregulation

## Abstract

Rising global temperatures driven by climate change have markedly increased the incidence of heat stress (HS) in monogastric livestock, posing critical challenges to animal welfare, health and productivity. This review provides a comprehensive analysis of the physiological and metabolic responses to HS in species such as poultry and swine. It details both the rapid thermoregulatory adaptations, including increased respiratory rate and peripheral vasodilation, and the sustained activation of the hypothalamic-pituitary-adrenal axis that elevates stress hormone levels. Chronic heat exposure disrupts immune function, induces oxidative stress via excessive reactive oxygen species production, and shifts metabolic balance from anabolic to catabolic processes, thereby impairing muscle development and compromising carcass composition. In response, a range of nutritional and management interventions, including antioxidant and osmolyte supplementation, dietary adjustments, enhanced housing designs, and genetic selection for heat tolerance, have been explored to mitigate these adverse effects. By integrating these multidisciplinary strategies, producers can improve animal welfare, sustain productivity, and preserve meat quality under escalating thermal challenges. The review further emphasizes the need for advanced monitoring technologies and precision livestock farming approaches to develop resilient, adaptive production systems in an era of climate uncertainty.

## Simple Summary

This review explores how heat stress (HS), worsened by climate change, affects the health and performance of monogastric livestock like poultry and swine. It explains how HS disrupts thermoregulation, hormone balance, immune response, and metabolism, leading to reduced growth, meat quality, and reproduction. The review also highlights differences between species, such as broilers and pigs, and presents strategies to manage HS, including nutrition, genetic selection, housing changes, and emerging technologies. Finally, it discusses the economic and practical implications of these interventions, stressing the importance of integrated and region-specific approaches for sustainable livestock production.

## 1. Introduction

Climate change is one of the most pressing challenges of our era, reshaping natural ecosystems and altering human activities [1]. Among its myriad impacts, rising global temperatures and more frequent heat waves exert significant pressure on animal agriculture [2]. Monogastric livestock, primarily poultry and swine, are particularly vulnerable to these thermal challenges due to their high metabolic rates and limited physiological mechanisms for effective heat dissipation [3,4]. As global warming advances, the incidence and severity of heat stress (HS) in these animals have increased dramatically, affecting both their health and productive performance [5], with consequences on metabolism, antioxidant balance, and genetic expression [6].

HS is defined as the physiological strain experienced by animals when environmental temperatures exceed their thermoneutral zone, disrupting the balance between heat production and heat loss [7]. Regarding mature poultry, the thermoneutral zone ranges from 18–24 °C for broilers and 18–22 °C for laying hens [8]. Concerning mature swine, the thermoneutral zone is 18–24 °C for growing and finishing pigs and 16–22 °C for gestating and lactating sows [9]. In monogastric species, such as poultry and swine, even slight deviations from optimal temperature conditions can trigger a cascade of physiological and behavioural responses aimed at maintaining homeostasis [3]. These include increased respiratory rate, peripheral vasodilation, and reduced activity and feed intake, all of which help to limit metabolic heat production and facilitate heat dissipation [4]. Although these responses are initially adaptive, chronic exposure to high temperatures overwhelms these mechanisms, leading to detrimental outcomes. Persistent HS disrupts endocrine balance by altering the secretion of key hormones, including cortisol, which is elevated as part of the stress response; thyroid hormones (T_3_ and T_4_), which are typically suppressed, reducing metabolic rate; and insulin, which may be altered due to changes in energy metabolism and feed intake. These hormonal fluctuations disrupt metabolic processes, impairing immune function and reducing overall welfare [6]. Additionally, oxidative stress and metabolic imbalances further exacerbate the physiological burden, compromising growth, reproduction, and productivity [10]. These effects make HS one of the most critical challenges in modern livestock production, requiring urgent mitigation strategies to sustain animal health and productivity.

HS’s dual impact on animal performance and health is a critical challenge for modern livestock production. From a performance standpoint, HS significantly reduces feed intake as an adaptive mechanism to lower metabolic heat production, leading to an energy deficit [10]. This energy shortfall impairs growth rates and muscle development and alters carcass composition, ultimately reducing meat production systems’ productivity and efficiency [11]. From a health perspective, prolonged exposure to HS disrupts homeostasis, increasing susceptibility to infections and stress-related diseases [12]. Additionally, HS disrupts redox homeostasis by increasing the production of reactive oxygen species (ROS), which leads to oxidative damage. Key biomarkers of this imbalance include malondialdehyde (MDA), a product of lipid peroxidation, as well as reductions in the activity of antioxidant enzymes such as superoxide dismutase (SOD), catalase, and glutathione peroxidase (GPx). This leads to oxidative stress that damages cellular components, compromises tissue integrity, and negatively impacts meat quality [13]. As a result, managing HS is not only essential for maintaining livestock productivity but also for safeguarding animal health and ensuring high-quality meat production.

Given the multifaceted challenges posed by HS, there is an urgent need to develop integrated strategies that mitigate both its physiological and production-related impacts. The primary objective of this review was to provide a comprehensive examination of the mechanisms by which HS impairs health and performance in monogastric livestock. Specifically, this review explores the systemic and cellular responses to HS, highlighting the roles of thermoregulatory, endocrine, and metabolic pathways [10]. Prolonged heat exposure shifts the balance from anabolic to catabolic processes, leading to compromised growth, muscle development, and meat quality [14]. Additionally, HS alters the gut microbiota composition in monogastric animals, resulting in reduced microbial diversity and a shift toward opportunistic pathogens. These changes impair nutrient absorption, disrupt energy metabolism, and compromise immune function, thereby exacerbating the physiological burden. For example, studies have shown that heat stress reduces the abundance of beneficial genera such as *Lactobacillus* and increases potentially harmful bacteria like Clostridium in poultry and swine [15,16]. A deeper understanding of these mechanisms is essential for developing targeted interventions that improve thermal resilience in livestock while maintaining productivity and animal welfare.

This review also evaluates both management and nutritional interventions to mitigate HS in monogastric livestock. Nutritional strategies, such as antioxidant supplementation (vitamin E, selenium, polyphenols) and osmolyte inclusion (betaine, taurine), help counteract oxidative damage and maintain cellular stability, while optimising nutrient formulations supports adequate nutrition despite reduced feed intake. Complementing these approaches, management practices like improved ventilation, cooling technologies, and optimised housing reduce the thermal load, and genetic selection offers a long-term solution by breeding animals that are more resilient to high temperatures. To support this analysis, an extensive literature search was performed across reference databases, such as PubMed, Scopus, and Web of Science, using the targeted keywords: “heat stress”, “monogastric physiology”, “performance”, “oxidative stress”, “nutritional interventions”, “antioxidants”, “poultry”, and “swine”. Priority was given to studies published within the last decade to ensure that the most recent advancements and findings are incorporated. By synthesising current research, this review elucidates the complex interplay between thermal stress, animal physiology, and animal health, ultimately underscoring the critical need for integrated and holistic strategies to enhance the sustainability and resilience of meat production systems in the face of escalating global temperatures.

## 2. Physiological and Metabolic Responses to Heat Stress

Exposure to elevated ambient temperatures sets in motion a cascade of physiological responses in monogastric livestock, aimed initially at protecting the animal by dissipating excess heat. However, when HS persists, these immediate defences can become overwhelmed, ultimately compromising health and productivity. The responses can be broadly categorized into thermoregulatory and behavioural adaptations, endocrine and immune disruptions, and metabolic reprogramming.

### 2.1. Thermoregulatory and Behavioural Adaptations

Monogastric animals employ a variety of rapid, short-term strategies to counteract increased environmental temperatures. One of the primary thermoregulatory mechanisms is an increase in respiratory rate; poultry, for instance, resort to panting as a means of facilitating evaporative cooling [17]. In swine, the limited functionality of sweat glands necessitates reliance on increased respiration, while both species benefit from peripheral vasodilation, which redirects blood flow to the skin surface to enhance heat dissipation and maintain core body temperature [18].

In parallel with these physiological changes, behavioural adaptations occur. Animals often reduce physical activity and seek shaded or cooler environments to limit additional heat production. A particularly well-documented adaptation is reduced feed intake. In poultry, feed intake may decrease by 20–25%, and in swine by 10–20%, under chronic HS conditions [19,20]. Although these adaptations are beneficial in the short term, their effectiveness declines as ambient temperatures remain high. With continued HS, these compensatory mechanisms become insufficient to prevent hyperthermia, leading to a state where the animal’s ability to regulate its body temperature is compromised. This failure of thermoregulation not only impairs immediate survival but also sets the stage for further systemic disturbances, including immune suppression and metabolic imbalances [21,22].

### 2.2. Endocrine and Immune Responses

Beyond the immediate physical and behavioural adaptations, HS exerts a profound impact on the endocrine and immune systems of monogastric livestock. A key physiological response to HS is the activation of the hypothalamic-pituitary-adrenal (HPA) axis, which serves as the primary mediator of the stress response [23]. In swine and poultry, acute HS stimulates the release of cortisol and corticosterone, respectively, which initially play a protective role by mobilising energy reserves through gluconeogenesis and lipolysis, ensuring sufficient energy availability for thermoregulatory mechanisms [24].

However, prolonged HS leads to sustained elevations in these stress hormones, pushing animals into a state of chronic stress. Studies have shown that corticosterone levels in broilers can increase by 1.5 to 2.5-fold under chronic heat stress compared to thermoneutral conditions [15,25]. Persistently high levels of cortisol and corticosterone disrupt normal metabolic and immune functions, shifting energy priorities away from growth and reproduction toward immediate survival [26]. Chronic HPA axis activation is associated with impaired protein synthesis, reduced muscle development, and inefficient nutrient utilisation, all of which negatively affect animal productivity [27].

Additionally, prolonged HS significantly weakens immune function, leaving animals more vulnerable to infections and inflammatory disorders. High cortisol and corticosterone levels suppress lymphocyte proliferation, antibody production, and cytokine secretion, impairing both innate and adaptive immunity [28]. This immunosuppressive state not only increases susceptibility to pathogens but also exacerbates systemic inflammation, compounding the negative effects of HS on overall health and welfare.

In addition to the immune response, the endocrine system plays a pivotal role in mediating physiological adaptations to HS in monogastric animals. Exposure to elevated temperatures activates the HPA axis, leading to increased secretion of stress-related hormones such as cortisol and adrenaline. Cortisol promotes hepatic gluconeogenesis and energy mobilisation to meet stress demands but may simultaneously suppress immune function and reduce growth performance [29]. Adrenaline enhances cardiovascular output by increasing heart rate and peripheral vasodilation, aiding heat dissipation [30]. Prolonged elevation of these hormones, however, can disrupt metabolic equilibrium, compromise reproductive performance, and elevate oxidative stress. For instance, recent studies in goats and pigs confirm that heat stress leads to elevated cortisol levels and altered thyroid hormone profiles, correlating with reduced productivity and thermoregulatory imbalance [31].

Thus, the endocrine and immune disruptions caused by HS highlight the urgent need for adaptive management strategies, including genetic selection for heat tolerance, nutritional interventions, and environmental modifications to mitigate the adverse effects on livestock productivity and health.

### 2.3. Metabolic Reprogramming

HS drives significant shifts in the metabolic processes of monogastric livestock, altering energy balance and nutrient utilisation. Under thermoneutral conditions, animals primarily rely on anabolic processes that support growth, tissue repair, and productivity. However, HS induces a metabolic shift from anabolism to catabolism, prioritising immediate survival over long-term growth [32].

A key driver of this metabolic shift is reduced feed intake, a common behavioural adaptation aimed at lowering metabolic heat production. This leads to an energy deficit, forcing the animal to mobilise stored energy sources, such as fat and muscle proteins, to meet immediate energy demands [11]. The consequence is increased lipolysis and protein degradation, indicative of a catabolic state. Additionally, chronic HS alters carbohydrate metabolism, leading to inefficient energy utilisation and exacerbating performance losses [33].

This metabolic reprogramming is further intensified by oxidative stress, a condition where excessive ROS are generated due to increased metabolic activity. When ROS production surpasses the capacity of the animal’s antioxidant defences, cellular structures, including proteins, lipids, and DNA, become susceptible to oxidative damage [34]. This oxidative stress reduces metabolic efficiency, perpetuating the cycle of catabolism and impairing nutrient utilisation [6].

Ultimately, the metabolic alterations induced by HS have far-reaching consequences. The depletion of energy reserves and the degradation of muscle proteins contribute to diminished growth rates, altered carcass composition, and lower meat quality [35]. These changes not only compromise animal health and welfare but also have economic implications, as reduced muscle accretion and poor tissue quality negatively impact meat production efficiency [16].

Prolonged heat exposure shifts the balance from anabolic to catabolic processes, leading to compromised growth, muscle development, and meat quality [36]. Additionally, HS alters the gut microbiota composition in monogastric animals, resulting in reduced microbial diversity and a shift toward opportunistic pathogens. These microbial changes, already well-documented in poultry and swine, impair nutrient absorption, disrupt energy metabolism, and compromise immune function [37,38]. These effects are integral to the overall metabolic burden of heat stress and should be considered alongside endocrine, oxidative, and nutritional factors when developing comprehensive mitigation strategies.

In summary, the physiological and metabolic responses to HS in monogastric livestock represent a complex interplay of adaptive and maladaptive mechanisms. Initial thermoregulatory and behavioural adaptations, such as increased respiratory rate, vasodilation, and reduced activity, provide crucial short-term relief from heat. However, when these measures are prolonged, they give way to deeper endocrine disruptions and a shift toward catabolic metabolism. The sustained activation of the HPA axis, with its attendant rise in stress hormones, impairs both immune function and tissue integrity, while metabolic reprogramming drives an energy deficit that compromises growth and repair processes (Table 1).

## 3. Impact of Heat Stress on Animal Health

HS imposes extensive challenges on animal health, extending beyond immediate physiological responses to systemic dysfunction. In monogastric species, prolonged exposure to elevated temperatures leads to a complex interplay of compromised immune function, increased oxidative stress, and behavioural alterations that collectively undermine animal welfare and productivity [12]. Understanding the full extent of these disruptions is critical for developing effective mitigation strategies and sustaining livestock productivity under increasing thermal pressures.

### 3.1. Immune Function and Disease Susceptibility

HS significantly suppresses the immune system of monogastric animals, increasing their vulnerability to infections and inflammatory disorders. A key driver of this immunosuppressive effect is the activation of the HPA axis, which triggers the secretion of cortisol in swine and corticosterone in poultry [6]. While these hormones initially help animals cope with stress by mobilising energy reserves through gluconeogenesis and lipolysis, their chronic elevation leads to profound immune suppression. Elevated glucocorticoid levels impair the proliferation and activity of lymphocytes, macrophages, and natural killer (NK) cells, thereby weakening the adaptive and innate immune responses [28].

HS also disrupts cytokine homeostasis, leading to an imbalance between pro-inflammatory (e.g., IL-1β, IL-6, TNF-α) and anti-inflammatory (e.g., IL-10) cytokines [26]. This imbalance triggers chronic low-grade inflammation, which not only weakens immune defences but also increases metabolic costs, further compromising animal growth and productivity [27].

Moreover, HS increases the risk of gastrointestinal and respiratory infections. Weakened gut barrier integrity and increased intestinal permeability facilitate the translocation of pathogenic bacteria and endotoxins, exacerbating systemic inflammation and infection rates [16]. HS is also associated with gut dysbiosis, defined as an imbalance in the intestinal microbiota, which further contributes to immune dysfunction. Under HS conditions, the abundance of beneficial microbes such as *Lactobacillus* decreases, while opportunistic pathogens like *E. coli* and *Clostridium perfringens* increase. This microbial imbalance impairs nutrient absorption and weakens mucosal defences [37,38]. Heat-stressed pigs and poultry have been shown to display higher susceptibility to diseases such as colibacillosis, necrotic enteritis, and respiratory syndromes [39].

Chronic HS also compromises the effectiveness of vaccines and other immune-related interventions. Elevated levels of stress hormones interfere with antigen presentation and antibody production, reducing the efficacy of vaccination programs [26]. This complicates disease management strategies, as heat-stressed animals often show weakened responses to both viral and bacterial pathogens.

In addition, heat-stressed animals exhibit impaired production of secretory immunoglobulin A (sIgA), which plays a crucial role in maintaining mucosal immunity in the respiratory and gastrointestinal tracts [27]. Reduced sIgA levels weaken the animal’s defence against enteric infections, further exacerbating gut health problems under HS conditions.

### 3.2. Stress Biomarkers and Health Indicators

Stress biomarkers provide critical insights into the physiological disruption caused by HS. Among the most widely recognised indicators are stress-related hormones, particularly cortisol, corticosterone, and adrenocorticotropic hormone (ACTH). These hormones are elevated as part of the activation of the HPA axis and serve as sensitive indicators of systemic stress responses. Elevated cortisol levels are consistently observed in heat-stressed animals and are associated with impaired growth, reduced feed efficiency, and heightened disease susceptibility [29].

In addition to hormonal changes, HS induces significant metabolic alterations, including elevated blood glucose levels driven by glucocorticoid-mediated gluconeogenesis and altered insulin sensitivity. These metabolic shifts are adaptive in the short term but may become detrimental if prolonged [31]. Other blood biochemical parameters also show marked alterations under HS. For instance, total protein and albumin concentrations tend to decrease, indicating compromised hepatic synthesis and increased catabolism. Blood urea nitrogen and creatinine levels may rise, reflecting impaired kidney function or dehydration-related stress, especially in swine. Elevated levels of liver enzymes such as alanine aminotransferase (ALT) and aspartate aminotransferase (AST) are also commonly observed, particularly in poultry, suggesting hepatic injury [40,41].

Oxidative stress is another major consequence of HS. Prolonged heat exposure leads to excessive production of reactive oxygen species (ROS), including superoxide anions (O_2_^−^), hydroxyl radicals (OH^−^), and hydrogen peroxide (H_2_O_2_), which exceed the capacity of endogenous antioxidant systems such as SOD, catalase, and GPx. Oxidative stress biomarkers, such as MDA, are elevated in heat-stressed animals, indicating increased lipid peroxidation and cellular damage [12,42].

HS also induces significant haematological and biochemical changes, such as reductions in red blood cell counts, haemoglobin concentrations, and haematocrit values, which collectively impair oxygen transport and energy metabolism. Concurrently, elevated levels of plasma non-esterified fatty acids (NEFA) and β-hydroxybutyrate suggest a shift toward catabolic metabolism, where animals rely on fat mobilisation for energy due to suppressed feed intake and heightened metabolic demands [43].

In addition, electrolyte imbalances, notably hyponatremia and hyperkalaemia, are commonly observed in animals under prolonged HS. These imbalances interfere with cellular function, manifesting in muscle weakness, cardiac arrhythmias, and neurological impairments. Disruption in sodium, potassium, and chloride homeostasis underscores the need to monitor both metabolic and ionic markers for effective HS mitigation [44].

It is important to note that these physiological changes can vary by species. For example, poultry often exhibit a sharper increase in AST and MDA levels under HS, while swine are more prone to elevated creatinine and urea due to their sensitivity to dehydration and renal stress. Recognising these interspecies differences can help tailor diagnostic and intervention strategies more effectively [40,45].

### 3.3. Behavioural and Welfare Considerations

Behavioural adaptations are among the earliest and most observable signs of HS in monogastric livestock. Reduced feed intake is a primary behavioural response, driven by the need to minimise metabolic heat production associated with digestion [22]. Heat-stressed pigs and poultry exhibit increased resting time, reduced exploratory behaviour, and increased panting or open-mouth breathing to facilitate evaporative cooling [21].

Altered feeding patterns under HS conditions lead to significant nutritional deficiencies, impairing growth and muscle development [46]. Reduced activity levels further contribute to muscle atrophy and increased fat deposition, diminishing overall carcass quality [47].

Heat-stressed animals also exhibit increased signs of discomfort and distress. Pigs and poultry tend to isolate themselves from other animals and avoid interaction. Increased aggression and pecking behaviour have also been documented in poultry under HS conditions [48].

Reproductive behaviour is also affected under HS conditions. Sows and hens exposed to prolonged HS show decreased fertility rates, reduced litter size, and increased embryonic mortality [6]. Male reproductive performance is similarly impaired, with reduced sperm count and motility documented in heat-stressed boars and roosters.

The impact of HS on animal health is extensive and multifaceted. Suppression of immune function and increased disease susceptibility highlight the vulnerability of heat-stressed animals. Elevated stress biomarkers, such as cortisol and indicators of oxidative damage (e.g., MDA), provide objective measures of physiological strain. At the same time, behavioural changes, including reduced feed intake, lethargy, and altered social interactions, further exacerbate these effects, signalling declining welfare (Table 2). Understanding these interconnected impacts is crucial for developing integrated nutritional, genetic, and management strategies aimed at bolstering the resilience of monogastric livestock and ensuring sustainable animal production in a warming world.

## 4. Effects of Heat Stress on Animal Performance

HS significantly compromises animal performance in monogastric livestock, affecting growth dynamics, carcass composition, and overall production efficiency. As ambient temperatures rise, the physiological adjustments and metabolic shifts induced by HS not only impair individual animal productivity but also pose broader economic challenges for the livestock industry. This section examines how HS impacts growth and productivity, alters carcass traits, and leads to economic repercussions.

### 4.1. Growth and Productivity

One of the most immediate and pronounced effects of HS in monogastric animals, such as poultry and swine, is a reduction in feed intake. Monogastric animals instinctively decrease their feeding activity in response to high ambient temperatures to minimise additional metabolic heat production [4]. This reduction in feed intake creates an energy deficit that limits the availability of essential nutrients required for growth and tissue repair, leading to slower weight gain and reduced overall growth rates [46].

In poultry, studies have shown that HS decreases daily feed intake by up to 15–20%, which translates into a significant drop in body weight gain and overall growth performance [6]. Broiler chickens exposed to chronic HS not only experience lower feed intake but also reduced nutrient absorption efficiency, further exacerbating the decline in growth rates. Swine experience similar effects, with reductions in average daily gain (ADG) of 10–15% under prolonged HS conditions [32].

HS also negatively affects feed conversion efficiency (FCE), a key performance indicator in livestock production. Under normal thermoneutral conditions, monogastric animals convert feed into body mass with relatively high efficiency. However, during HS, energy that would typically be directed toward growth is instead diverted toward thermoregulatory processes such as increased respiratory rate and vasodilation. This metabolic shift results in poorer FCE, meaning that more feed is required to achieve the same weight gain, ultimately increasing production costs [11].

In laying hens, HS reduces not only feed intake but also egg production and egg quality. Mechanistically, this is due to the decreased intestinal absorption of calcium and the suppression of vitamin D_3_ synthesis, both of which are essential for calcium metabolism and eggshell formation. Heat stress also affects parathyroid hormone (PTH) regulation, further impairing calcium mobilisation from bone reserves. As a result, eggs produced under HS conditions tend to have thinner shells and higher breakage rates [6]. Similarly, in growing pigs, reduced feed intake causes lower carcass yields and decreased muscle mass, further degrading meat quality [32].

HS significantly impacts protein metabolism. Under thermoneutral conditions, animals prioritise protein synthesis for muscle development and tissue repair. However, HS triggers a metabolic shift from anabolism to catabolism, where muscle protein breakdown exceeds protein synthesis [11].

Elevated circulating cortisol levels under HS conditions activate proteolysis and inhibit protein synthesis in skeletal muscle. Studies in swine have shown that HS reduces the expression of genes involved in muscle growth and repair while increasing the activity of proteolytic enzymes such as calpains and caspases [6]. This process results in muscle atrophy, reduced muscle fibre size, and impaired muscle accretion. Importantly, HS also reduces circulating levels of insulin-like growth factor 1 (IGF-1), a hormone critical for muscle development and protein synthesis. Lower IGF-1 concentrations are associated with reduced muscle cell proliferation and decreased lean tissue growth, particularly in swine and broilers [52].

In poultry, heat-stressed broilers exhibit decreased breast muscle yield due to a reduction in muscle fibre hypertrophy and increased muscle proteolysis [47]. This leads to compromised carcass quality and diminished market value. Furthermore, the reduction in muscle accretion contributes to poorer feed efficiency and lower overall productivity. In addition to direct muscle degradation, HS impairs post-translational modifications of muscle proteins, altering meat texture and water-holding capacity. This contributes to increased drip loss and poorer meat quality in both swine and poultry [53].

Thyroid hormones, specifically triiodothyronine (T_3_) and thyroxine (T_4_), are also significantly affected by heat stress. Under elevated temperatures, circulating levels of T_3_ often decline, contributing to a reduction in basal metabolic rate as an adaptive response to minimise internal heat production. This endocrine adjustment, while protective, also slows growth and reduces energy availability for productive functions, particularly in high-growth breeds such as broilers [54].

HS also alters nutrient partitioning by increasing the reliance on lipid metabolism for energy. Under thermoneutral conditions, monogastric animals primarily derive energy from carbohydrates and protein metabolism. However, during HS, increased metabolic heat production from carbohydrate oxidation creates an additional thermal burden, prompting a metabolic shift toward lipolysis (fat metabolism) to reduce heat load [32].

In heat-stressed pigs, plasma levels of NEFAs and ketone bodies, such as β-hydroxybutyrate, increase, indicating elevated lipid mobilisation. This metabolic shift reflects the animal’s adaptation to energy deficits caused by reduced feed intake and increased thermoregulatory demands [11].

The increased reliance on lipid metabolism results in greater fat deposition and reduced muscle-to-fat ratios. In poultry, heat-stressed broilers exhibit higher abdominal fat levels and lower breast muscle yield, leading to undesirable carcass composition and poorer processing efficiency [47]. In swine, increased backfat thickness and reduced muscle mass further decrease carcass value and market appeal.

Increased fat deposition under HS conditions also contributes to poorer meat quality. Higher intramuscular fat content alters the texture and flavour profile of meat while increasing the susceptibility to oxidative rancidity, further degrading sensory properties and shelf life [53].

It is also important to recognise that the economic impact of these production losses varies significantly across regions. For example, livestock producers in developing countries, who often lack access to cooling systems, nutritional supplements, and veterinary care, are likely to experience more severe performance and financial losses under HS. In contrast, producers in developed regions may be better equipped to mitigate these impacts through advanced infrastructure and precision farming technologies. Including this perspective enhances the global relevance of the discussion on HS and its consequences [55].

### 4.2. Carcass Composition and Production Metrics

HS not only impairs growth performance but also significantly impacts carcass composition and production metrics in monogastric animals such as poultry and swine [56]. The physiological disturbances triggered by HS lead to metabolic imbalances that compromise muscle development, fat deposition, and overall carcass quality. These changes are critical because they directly influence the market value and consumer acceptance of meat products derived from heat-stressed animals.

Under prolonged HS conditions, the shift from anabolic to catabolic metabolism becomes more pronounced. In thermoneutral conditions, monogastric animals prioritize muscle protein synthesis to support lean muscle growth. However, HS triggers the activation of the HPA axis, leading to increased circulating cortisol levels. Cortisol promotes muscle protein degradation while simultaneously inhibiting protein synthesis, resulting in muscle atrophy and lower lean muscle mass [46].

This catabolic state is characterised by increased activity of proteolytic enzymes, including calpains and caspases, which break down muscle proteins into free amino acids. In swine, heat-stressed animals exhibit reduced loin muscle area and increased muscle fat content, contributing to poorer carcass quality and reduced market value. In poultry, similar effects are observed, with heat-stressed broilers showing lower breast muscle yield and increased abdominal fat content [47].

HS also affects muscle fibre composition. Fast-twitch muscle fibres (type II) are more susceptible to heat-induced atrophy than slow-twitch fibres (type I). This imbalance leads to poorer meat texture and increased variability in muscle quality [53]. Swine exposed to HS show increased muscle fibre fragmentation and reduced cross-sectional muscle fibre area, leading to reduced tenderness and increased drip loss.

HS also modifies muscle structure and biochemical composition, directly influencing meat texture, tenderness, and water-holding capacity. Muscle fibres exposed to prolonged HS exhibit increased sarcomere shortening and increased connective tissue cross-linking, both of which contribute to tougher meat texture [47].

In swine, reduced muscle glycogen content under HS conditions decreases *post-mortem* lactic acid production, resulting in higher ultimate pH and reduced meat tenderness. Reduced glycogen reserves also lead to darker meat colour and increased susceptibility to oxidative rancidity [46].

In poultry, the impact of HS on meat texture and tenderness is equally significant. Heat-stressed broilers produce meat with increased toughness, lower water-holding capacity, and greater shear force. PSE (pale, soft, and exudative) meat is a common outcome of HS in poultry, characterised by low muscle pH, increased drip loss, and reduced juiciness. Increased oxidative damage and protein denaturation under HS conditions further compromise muscle integrity, reducing sensory quality and consumer acceptance [53].

The tenderness of poultry meat is directly influenced by changes in muscle fibre structure and protein integrity. Heat-stressed broilers exhibit increased expression of heat shock proteins (HSPs), which are involved in cellular stress response and muscle repair. However, the overexpression of HSPs interferes with normal muscle development and post-mortem tenderization, contributing to increased meat toughness and lower overall quality [6].

The oxidative stress induced by HS further degrades carcass quality. Elevated body temperatures increase the production of ROS, which target cellular lipids, proteins, and DNA, leading to oxidative damage and compromised muscle integrity. Lipid peroxidation results in the formation of MDA, a marker of oxidative stress that contributes to off-flavours and reduced meat shelf life [53].

HS-induced oxidative stress also damages muscle protein structures, leading to increased protein oxidation and altered muscle colour. Oxidised myoglobin forms metmyoglobin, which gives meat a brownish hue, reducing consumer appeal and market value. Increased protein oxidation also reduces muscle water-holding capacity, contributing to higher drip loss and poorer meat texture [6].

HS-induced metabolic changes increase the deposition of abdominal fat in poultry and backfat in swine. This metabolic shift reflects the animal’s adaptive strategy to rely more heavily on lipid metabolism under heat-stressed conditions. Reduced muscle accretion combined with increased lipogenesis results in a higher fat-to-lean ratio, reducing overall carcass value and marketability [46].

In poultry, abdominal fat levels increase by up to 20% under prolonged HS conditions, reducing overall carcass yield and increasing processing costs. Higher fat deposition increases the risk of oxidative rancidity and reduces meat shelf life [47]. In swine, increased backfat thickness reduces lean meat yield and lowers the market value of carcasses.

Excess fat deposition also affects carcass conformation and processing efficiency. Leaner carcasses are generally preferred in the market due to higher muscle yield and reduced fat trimming costs. Therefore, HS-induced fat deposition represents a significant economic loss for producers.

### 4.3. Reproductive Performance

HS profoundly impacts reproductive performance in monogastric livestock, including swine and poultry, through a combination of endocrine disruption, impaired gametogenesis, and increased embryonic mortality. Reproduction is a highly energy-dependent process, and the metabolic and hormonal disturbances caused by HS compromise the reproductive axis at multiple levels, from gamete production to fertilisation and early embryonic development [57]. The resulting decline in reproductive efficiency translates into lower farrowing and hatching rates, smaller litter sizes, and increased production costs.

Prolonged HS in female monogastric livestock, particularly sows, reduces fertility rates and shortens the duration of oestrus [58]. This disruption is primarily linked to increased circulating cortisol levels, which interfere with the hypothalamic-pituitary-gonadal (HPG) axis. Elevated cortisol inhibits gonadotropin-releasing hormone (GnRH) secretion from the hypothalamus, leading to reduced secretion of luteinizing hormone (LH) and follicle-stimulating hormone (FSH) from the anterior pituitary. Since LH and FSH are critical for follicular development and ovulation, the suppression of these hormones results in poor follicular growth, delayed ovulation, and reduced conception rates [32].

HS also increases the production of prostaglandin F2α (PGF2α) in the endometrium, which leads to early regression of the corpus luteum (CL) and shortened luteal phases. Reduced progesterone production from the CL impairs the ability of the uterus to support embryo implantation, contributing to higher embryonic mortality rates [32].

Studies in swine have shown that sows exposed to HS during the early luteal phase experience up to a 30% reduction in conception rates and a 20–25% increase in embryonic mortality [51]. Embryos that survive early HS often display impaired development and lower implantation success due to oxidative damage and metabolic stress in the uterine environment.

In poultry, HS disrupts laying patterns and egg production. Heat-stressed hens lay fewer eggs, and the eggs produced under HS conditions are often smaller, have thinner shells, and exhibit lower hatchability. Calcium metabolism is impaired under HS, leading to lower calcium availability for eggshell formation, which increases the likelihood of shell deformation and cracking. Reduced yolk quality and lower egg mass further compromise the economic value of heat-stressed eggs [6].

Male reproductive performance is equally affected by HS, with reductions in sperm quality, testicular size, and overall reproductive efficiency. Spermatogenesis is highly sensitive to temperature, and even modest increases in scrotal temperature (by 1–2 °C) can impair sperm production and testicular function. HS triggers testicular oxidative stress, which leads to lipid peroxidation of sperm membranes and increased DNA fragmentation, reducing sperm motility and viability [51].

Studies in boars exposed to HS have reported: up to a 30% reduction in sperm concentration; a 40% decline in sperm motility; increased sperm morphological abnormalities (e.g., coiled tails, detached heads); and reduced seminal volume and lower ejaculate quality. Testicular size is also significantly reduced under HS conditions. This shrinkage is linked to reduced testosterone synthesis, impaired Leydig cell function, and increased testicular apoptosis. Lower testosterone levels impair libido and mating performance, reducing overall reproductive output [32].

In poultry, the impact of HS on male reproductive performance includes reduced semen volume, lower sperm motility, and decreased sperm viability. Testicular atrophy and increased testicular apoptosis under HS conditions have been linked to reduced expression of heat shock proteins (HSPs), which are critical for protecting sperm cells from oxidative damage [6].

HS alters the delicate balance of reproductive hormones, which further compounds the decline in reproductive performance. Elevated cortisol levels inhibit GnRH secretion, reducing circulating LH and FSH levels and impairing follicular and sperm development. Additionally, increased prolactin levels under HS conditions suppress ovarian activity and reduce oestrous cyclicity in female livestock [32].

In sows, HS reduces progesterone levels due to impaired CL function, leading to shorter oestrous cycles and reduced implantation success. In boars, reduced testosterone production under HS conditions impairs libido and sperm quality, further reducing reproductive efficiency [51].

In poultry, increased circulating corticosterone under HS conditions reduces the production of androgens and oestrogens, disrupting egg production and lowering fertility rates. Reduced luteinizing hormone (LH) secretion and impaired follicular development contribute to delayed ovulation and smaller clutch sizes [6].

HS also affects early embryonic development by reducing uterine blood flow and increasing uterine temperature. Elevated maternal body temperatures increase embryonic mortality by disrupting cellular homeostasis and increasing oxidative stress. In swine, embryonic losses under HS conditions can reach up to 40% during early gestation, with surviving embryos exhibiting impaired growth and lower birth weights [32].

In poultry, HS during incubation increases embryonic mortality rates and reduces hatchability. HS disrupts eggshell gas exchange, reduces oxygen availability, and increases embryonic hypoxia. Surviving chicks are often smaller, less viable, and more prone to early post-hatch mortality [6].

### 4.4. Economic Implications

HS imposes substantial economic burdens on monogastric livestock industries, particularly in swine and poultry systems. Decreased feed intake, impaired feed conversion efficiency (FCE), and inferior carcass composition all contribute to reduced productivity and profitability. For example, HS can reduce average daily gain in swine by up to 20% and increase FCE by 10–15%, directly elevating feed costs for each unit of weight gain [12].

In poultry, HS can lower growth rates by 15–25% and increase production cycle duration, thus requiring additional resources such as feed, water, and energy to reach market weight. Laying hens also show reduced feed intake and egg production (10–15%), with eggs of lower quality and higher breakage rates, which diminishes their market value [44].

Carcass composition suffers under HS as well. In poultry, fat deposition may increase by 20%, decreasing lean yield and shelf life due to oxidative rancidity. Similarly, swine exposed to HS exhibit increased backfat and reduced loin muscle area, negatively affecting market pricing and consumer preference [43].

Reproductive efficiency also declines markedly. HS can reduce sow conception rates by up to 30% and increase embryonic mortality by 20–25%. In poultry, fertility declines and early embryonic mortality rise, affecting hatchability and overall production output [12].

Beyond direct production impacts, HS introduces market instability and raises operational costs. Cooling infrastructure, nutritional supplementation (e.g., electrolytes and antioxidants), and veterinary care represent major financial commitments. Mortality rates can rise by 5–10%, leading to further costs in disposal and animal replacement [42].

The global economic toll of HS in livestock is estimated to exceed $1 billion annually. However, integrated mitigation strategies offer cost-effective solutions. Combining environmental modifications (e.g., ventilation, misting) with nutritional support can reduce ambient temperatures by 3–5 °C, improve FCE by up to 10%, and reduce mortality by 8–12% [36].

Genetic selection for heat-tolerant breeds further strengthens resilience and long-term profitability. Although upfront investment is high, the return in terms of improved animal performance, reduced mortality, and greater market consistency validates a multi-pronged approach to HS mitigation.

**Figure 1:**
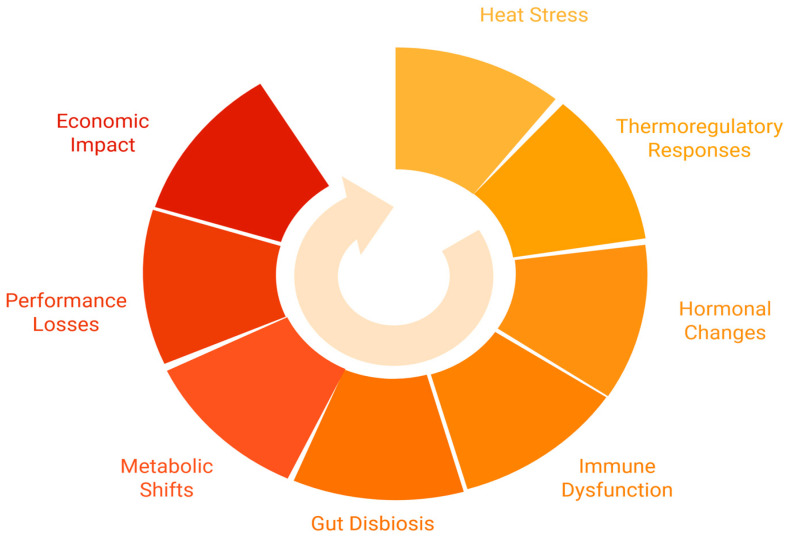
Circular network diagram illustrating the cascading effects of heat stress in monogastric livestock, from initial thermoregulatory responses to broader metabolic shifts and economic impact.

Table 3 outlines the key impacts of HS on growth, carcass composition, reproductive performance, and economic outcomes. The table also details the consequences of these impacts and includes relevant references to support the findings. In addition, Figure 1 provides a visual summary of the cascade of physiological, metabolic, and economic effects of heat stress in monogastric animals.

## 5. Management and Nutritional Interventions for Enhancing Health and Performance

Mitigating the detrimental effects of HS in monogastric livestock requires a multifaceted approach that integrates nutritional, environmental, and genetic strategies. By combining these interventions, producers can enhance animal resilience, sustain growth performance, and improve overall meat quality even under challenging thermal conditions [59].

### 5.1. Nutritional Strategies

Nutritional interventions are among the most effective and practical solutions for mitigating HS effects in livestock. One of the primary nutritional strategies is antioxidant supplementation, as oxidative stress is a major consequence of prolonged heat exposure. Vitamin E, selenium, and polyphenols enhance the animal’s endogenous antioxidant defences, helping to neutralise ROS and protect cellular integrity [60]. These compounds play a crucial role in preserving muscle tissue, improving immune responses, and maintaining meat quality.

In addition to antioxidants, osmolytes such as betaine and taurine have shown effectiveness in improving HS resilience. However, their efficacy and physiological roles can differ between species and even among strains. For instance, betaine has been shown to enhance osmotic balance, carcass leanness, and thermoregulation in both poultry and swine, but its effects are often more pronounced in broilers due to their higher growth rates and metabolic demands. In layers, betaine also improves eggshell quality by supporting calcium metabolism under stress conditions. Taurine, known for its antioxidant and anti-inflammatory functions, has demonstrated effectiveness in improving growth and gut integrity in broilers, while in swine, it may exert stronger immunomodulatory and hepatic protective effects, particularly during prolonged thermal exposure [61].

Adjusting nutrient formulations is also critical; optimising the protein-to-energy ratio and increasing dietary energy density compensates for reduced feed intake, ensuring that animals continue to receive essential nutrients despite lower consumption. For example, broilers may benefit more from increased dietary fat to boost energy density, whereas swine diets may prioritise amino acid balance to preserve lean growth during HS [62].

Functional feed additives, such as probiotics, prebiotics, and phytochemicals, further contribute to gut health and immune modulation. These additives enhance nutrient absorption, stabilise gut microbiota, and reduce inflammation, mitigating the negative metabolic effects of HS [16]. By maintaining a balanced gastrointestinal ecosystem, these interventions help sustain growth performance and prevent metabolic disturbances associated with prolonged exposure to high temperatures.

### 5.2. Environmental and Housing Modifications

Modifications to the housing environment are essential for reducing the thermal load on monogastric animals. Cooling systems, including misting, evaporative cooling pads, and fans, effectively lower ambient temperatures and create a more stable microclimate within livestock facilities [63]. Improved ventilation and air circulation also help dissipate heat efficiently, preventing localised overheating.

Housing design plays a key role in minimising HS. Incorporating shaded areas, reflective roofing materials, and insulation lowers internal temperatures, reducing the animal’s need for excessive thermoregulatory energy expenditure [64]. These modifications not only enhance animal comfort but also support nutrient partitioning toward growth rather than survival-based metabolic functions. Furthermore, reducing heat accumulation within housing facilities minimises the build-up of harmful gases and pathogens, thereby improving overall livestock health and performance.

Emerging technologies such as Precision Livestock Farming (PLF) offer innovative tools for continuous environmental and animal monitoring. PLF systems use sensors and data analytics to assess thermal stress in real time, allowing producers to adjust ventilation, feeding, and cooling interventions more effectively and dynamically [65].

### 5.3. Genetic and Breeding Approaches

Long-term mitigation of HS can be achieved through genetic selection for heat tolerance. Advances in genomic technologies have identified key genetic markers associated with thermoregulation, enabling breeding programs to select animals with superior heat resilience [66].

Genetic selection for heat tolerance does not come at the expense of productivity; modern breeding strategies aim to combine thermal resilience with high growth rates, efficient feed conversion, and superior meat quality. This synergistic approach ensures that genetic improvements not only enhance heat resilience but also maintain economic viability and meat industry standards [67,68].

In poultry, cutting-edge hatchery strategies such as in ovo feeding and thermal manipulation during embryonic development have shown promise in improving thermotolerance post-hatch. These interventions can enhance stress resilience, immune function, and growth performance without altering genetic structure, offering practical, scalable options for producers in heat-prone regions [69,70].

### 5.4. Integrated Management Approaches

Although individual strategies, nutritional, environmental, and genetic, offer distinct advantages, the most effective solution is an integrated management system that combines these interventions. Addressing HS on multiple levels allows producers to develop a holistic strategy that promotes animal welfare, optimises production efficiency, and reduces economic losses [71]. For example, combining nutritional supplementation with environmental cooling ensures that, even if an animal’s feed intake declines, its diet remains optimised to deliver maximum benefits [61]. Similarly, genetic selection for heat-tolerant traits, coupled with improved housing design, reduces the physiological burden imposed by HS. By integrating these strategies, producers create a robust framework that enhances livestock resilience and sustains productivity in the face of climate change.

However, implementing an integrated management system requires continuous monitoring and adaptation to production-specific needs. Regular assessment of performance metrics, stress biomarkers, and welfare indicators is crucial for fine-tuning interventions. Effective collaboration among nutritionists, geneticists, veterinarians, and farm managers ensures that customised solutions are developed to address the unique challenges posed by HS in different production environments.

Beyond the farm level, broader system-level adaptations are essential. These include regional supply chain adjustments to minimise stress during transport, government-supported investment in climate-resilient infrastructure, and development of policy frameworks that incentivise HS mitigation practices. Tailoring strategies to regional climate conditions and economic capabilities, especially in developing countries, ensures practical implementation and maximises long-term benefits across livestock systems globally [51,72].

Mitigating the negative effects of HS in monogastric livestock demands a comprehensive and multi-pronged approach. By implementing antioxidant and osmolyte supplementation, optimising dietary formulations, enhancing housing design, and selecting for heat-tolerant genetics, producers can significantly reduce the detrimental impact of HS (Table 4). This holistic strategy improves animal health and welfare, maintains growth performance, and preserves meat quality, ensuring the long-term sustainability and profitability of livestock production in the face of rising global temperatures.

## 6. Practical Implications and Economic Considerations

While understanding the physiological, metabolic, and molecular responses to HS is essential, translating these insights into effective on-farm practices is equally critical. Implementing mitigation strategies for HS in monogastric livestock presents both challenges and opportunities, requiring a balance between animal welfare improvements and financial viability. This section explores HS interventions’ real-world constraints and economic implications while emphasising the importance of integrated management approaches.

### 6.1. Implementation Challenges and Opportunities

Adopting HS mitigation strategies in commercial livestock operations is not without challenges. Production systems vary widely in terms of farm size, housing design, and available resources, requiring tailored interventions that align with specific regional climates and economic conditions [73]. For instance, advanced cooling systems such as evaporative cooling pads and misting technologies have demonstrated effectiveness in mitigating HS, yet the high upfront investment and operational costs may limit accessibility, particularly for small-scale producers. Similarly, nutritional strategies, including antioxidant supplementation and osmolyte inclusion, depend on precise feed formulations and consistent feed quality, factors that can be difficult to standardise in diverse production environments [12].

It is also important to consider the potential unintended consequences of these mitigation strategies. Nutritional interventions, while effective, may increase feed costs and alter primary feed formulations, potentially affecting feed availability or compatibility with other dietary components. Similarly, environmental modifications, particularly the installation of high-capacity cooling systems, carry substantial energy demands, which not only increase operational costs but also contribute to the overall environmental footprint of the farm. These trade-offs should be factored into decision-making when selecting and implementing mitigation technologies [74,75].

Despite these challenges, opportunities for innovation are emerging. Precision livestock farming technologies, such as real-time biometric monitoring systems, automated climate control, and precision feeding, allow for early detection of HS and enable timely adjustments to environmental and nutritional management. These technologies enhance animal welfare and productivity while potentially reducing long-term operational costs [16]. The adoption of such innovations, particularly in large-scale operations, can help mitigate the economic burden of HS by improving production efficiency and reducing losses.

### 6.2. Economic Impacts and Cost-Benefit Analysis

The economic consequences of HS extend beyond immediate production losses, such as reduced growth rates and impaired feed conversion efficiency. Long-term exposure to elevated temperatures can affect carcass composition, meat quality, and overall market value, influencing consumer acceptance and industry competitiveness [53].

Investments in nutritional interventions, including dietary antioxidants, osmolytes, and functional feed additives, may increase short-term feed costs. However, these expenditures are often offset by improvements in growth performance, feed efficiency, and meat quality. Similarly, capital-intensive environmental modifications, such as enhanced ventilation, advanced cooling systems, and optimized housing designs, require substantial initial investments but can lead to lower mortality rates, reduced veterinary costs, and improved overall productivity [63].

A cost-benefit analysis must consider both direct production metrics and broader economic implications. While high-tech interventions may not be viable for all farms, integrated management strategies that combine nutritional, environmental, and genetic approaches often yield synergistic benefits. For instance, farms implementing both dietary enhancements and advanced cooling systems have reported more stable performance and lower production losses during heat waves [76]. The economic viability of these strategies is ultimately shaped by a combination of local climatic conditions, farm-specific characteristics, and the availability of supportive technologies.

### 6.3. Welfare Outcomes and Integrated Management Approaches

Improving animal welfare under HS conditions is not only an ethical obligation but also a critical factor in sustainable livestock production. Welfare improvements, such as reduced stress behaviours, lower disease incidence, and optimized physiological parameters, are closely linked to higher productivity and improved product quality [73].

An integrated management strategy that simultaneously targets nutrition, housing, and genetic selection offers the most comprehensive approach to enhancing resilience against HS. For instance, optimizing diets with antioxidants and osmolytes while simultaneously improving housing ventilation and cooling infrastructure ensures that animals can maintain optimal metabolic function and growth performance even under challenging thermal conditions [77]. Genetic selection for heat-tolerant breeds further reinforces these interventions, enabling greater adaptability and improved long-term sustainability.

Successful implementation of such comprehensive strategies requires collaboration among nutritionists, geneticists, veterinarians, and farm managers. A coordinated approach ensures that tailored interventions are developed to address the specific challenges posed by HS across diverse production environments.

### 6.4. Future Directions in Practical Applications

Looking ahead, emerging technologies and precision farming innovations hold immense promise for further enhancing HS mitigation strategies. Sensor technology and real-time data analytics are paving the way for precision livestock farming, where continuous monitoring of environmental conditions and animal health indicators enables dynamic, adaptive management decisions. These tools include AI-driven platforms capable of detecting early signs of heat stress, predicting risk scenarios, and optimising responses such as targeted cooling or nutritional adjustments. By integrating artificial intelligence with farm management systems, producers can improve decision-making accuracy and responsiveness under fluctuating thermal conditions [73].

Additionally, advancements in nutrigenomics and precision nutrition will allow for more personalised dietary formulations that adapt to the specific metabolic demands of animals under different thermal conditions. Tailored feeding strategies informed by real-time physiological and environmental data can help maintain animal welfare and productivity even during extreme heat events [78].

We have also identified epigenetic adaptations to HS as an emerging and understudied area of research. Heat-induced epigenetic modifications, such as DNA methylation, histone modification, and non-coding RNA activity, may contribute to transgenerational resilience. While current understanding in monogastric livestock remains limited, this field holds potential for developing long-term adaptation strategies through selective breeding or targeted interventions [57].

With ongoing genetic research, breeding programs can further refine selection criteria for heat-tolerant livestock, ensuring that future generations are better equipped to withstand climatic challenges [66].

The practical implications and economic considerations of HS mitigation are central to bridging the gap between scientific knowledge and real-world applications. By addressing implementation challenges, conducting thorough cost-benefit analyses, and promoting integrated management approaches, producers can effectively mitigate the adverse effects of HS. These strategies enhance animal welfare and performance while ensuring the long-term economic sustainability of monogastric livestock production in an era of rising global temperatures [77,79].

## 7. Conclusions and Future Research

HS is a big challenge for monogastric livestock, undermining animal welfare, productivity, and meat quality. This review has elucidated how elevated temperatures disrupt thermoregulation, trigger chronic activation of the HPA axis, and shift metabolism from anabolic to catabolic processes. Such physiological disturbances, evidenced by reduced feed intake, impaired muscle development, and deteriorated carcass composition, translate into significant economic losses for the livestock industry.

While immediate adaptive responses like increased respiratory rate, vasodilation, and behavioural modifications provide short-term relief, prolonged exposure overwhelms these mechanisms, leading to immune suppression and oxidative damage. Nutritional interventions, when combined with environmental improvements and genetic selection for heat tolerance, offer promising avenues for mitigating these effects. Yet, the variability in responses across different production systems highlights the urgent need for tailored, integrated management strategies.

Looking forward, future research must prioritise longitudinal studies that capture the cumulative impacts of chronic HS and refine species-specific intervention protocols. The integration of advanced monitoring technologies, such as real-time sensors and predictive analytics, with emerging fields like nutrigenomics and precision livestock farming will be key to developing adaptive, data-driven solutions. Ultimately, fostering interdisciplinary collaboration among nutritionists, geneticists, veterinarians, farm managers, and policymakers is critical to translating these scientific insights into practical, resilient production systems.

## Figures and Tables

**Table 1:** Cascade of physiological and metabolic responses to heat stress.

Stage	Key Process	Outcomes	References
Immediate Response	- Increased respiratory rate and panting in poultry; elevated respiration in swine - Peripheral vasodilation - Behavioural changes (seeking shade, reduced activity)	Rapid heat dissipation - Temporary reduction in metabolic heat production	[17,18]
Endocrine Activation	- Activation of the HPA axis - Elevated cortisol (swine) or corticosterone (poultry)	- Mobilization of energy via gluconeogenesis and lipolysis	[23]
Metabolic Reprogramming	- Shift from anabolic to catabolic processes - Reduced feed intake leading to increased lipolysis and protein degradation	- Impaired muscle development - Reduced growth performance	[11,32]
Immune Suppression & Oxidative Stress	- Suppression of immune cell function - Overproduction of reactive oxygen species - Elevated oxidative stress biomarkers (e.g., MDA, reduced antioxidant enzymes)	- Increased susceptibility to infections - Cellular and tissue damage	[12,28,34]

**Table 2:** Summary of the impact of heat stress on animal health, highlighting the effects on immune function, stress biomarkers, and behavioural changes.

Category	Effect	Consequence	References
Immune Function and Disease Susceptibility	Suppression of adaptive and innate immunity due to elevated cortisol/corticosterone levels	Weakened immune defences, higher disease vulnerability	[6,28]
Immune Function and Disease Susceptibility	Imbalance between pro-inflammatory (IL-1β, IL-6, TNF-α) and anti-inflammatory cytokines (IL-10)	Chronic low-grade inflammation and metabolic cost	[26,27]
Immune Function and Disease Susceptibility	Increased susceptibility to gastrointestinal and respiratory infections	Higher infection rates and impaired gut barrier integrity	[16,39]
Stress Biomarkers and Health Indicators	Elevated cortisol levels reflecting metabolic and immune stress	Reduced growth, impaired feed efficiency, and higher disease risk	[26,28]
Stress Biomarkers and Health Indicators	Oxidative stress due to excessive ROS production leads to lipid peroxidation and cellular damage	Increased MDA levels and oxidative damage	[10,49]
Stress Biomarkers and Health Indicators	Reduced red blood cell counts, haemoglobin levels, and haematocrit values	Impaired oxygen transport and metabolic function	[49]
Stress Biomarkers and Health Indicators	Electrolyte imbalance (hyponatremia, hyperkalemia) leading to muscle weakness and cardiac issues	Impaired neural function and muscle weakness	[49]
Behavioural and Welfare Considerations	Reduced feed intake to minimise metabolic heat production	Poor growth and reduced muscle development	[22,50]
Behavioural and Welfare Considerations	Increased resting time, panting, and open-mouth breathing for thermoregulation	Increased respiratory alkalosis and acid-base imbalance	[21]
Behavioural and Welfare Considerations	Altered feeding patterns leading to nutritional deficiencies and impaired growth	Poor carcass quality and reduced muscle mass	[47,51]
Behavioural and Welfare Considerations	Increased aggression and social isolation, particularly in poultry	Lower animal welfare and higher stress levels	[48]

**Table 3:** Summary of the effects of heat stress on monogastric livestock, detailing the impacts on growth, carcass composition, reproductive performance, and economic outcomes.

Aspect	Impact	Consequence	References
Growth and Productivity	Reduced feed intake due to heat-induced metabolic stress, leading to lower energy availability and slower weight gain.	Lower body weight gain, increased feed costs, and longer production cycles.	[6,32]
Growth and Productivity	Increased oxidative stress reduces nutrient absorption efficiency and muscle development, limiting growth rates.	Reduced growth performance, increased feed conversion ratio (FCR), and increased production costs.	[11,32]
Carcass Composition and Production Metrics	Increased fat deposition and reduced lean muscle mass due to increased lipolysis and reduced muscle protein synthesis.	Reduced tenderness, increased drip loss, and poor meat texture, decrease consumer acceptance and market competitiveness.	[47,53]
Carcass Composition and Production Metrics	Altered muscle fibre composition reduces meat texture, increases drip loss, and reduces water-holding capacity.	Lower water-holding capacity and increased fat content increase processing costs and reduce product quality.	[6,53]
Reproductive Performance	Reduced fertility due to impaired secretion of gonadotropin-releasing hormone (GnRH), leading to lower ovulation rates.	Lower conception rates and reduced litter size, increasing replacement costs and reducing production efficiency.	[32,51]
Reproductive Performance	Increased embryonic mortality and poor sperm quality due to heat-induced oxidative stress and testicular dysfunction.	Reduced sperm concentration, motility, and increased sperm abnormalities, leading to lower fertility rates.	[32,51]
Economic Implications	Higher production costs due to increased cooling system requirements and higher veterinary costs.	Higher input costs, increased mortality rates, and longer production cycles reduce profitability.	[51,53]
Economic Implications	Reduced market value due to increased fat deposition, lower muscle yield, and poorer meat texture, reducing consumer demand.	Increased processing losses, reduced consumer demand, and price instability reduce overall profitability.	[47,53]

**Table 4:** Overview of management and nutritional interventions with the corresponding benefits.

Category	Intervention	Primary Benefit	References
Nutritional	Antioxidant supplementation (vitamin E, selenium, polyphenols)	Reduces oxidative stress, protects cell membranes, and preserves muscle quality	[60]
Nutritional	Osmolyte inclusion (betaine, taurine)	Stabilizes cellular structures and maintains osmotic balance, enhancing resilience under heat stress	[61]
Nutritional	Optimized nutrient formulation (adjusting protein-to-energy ratio)	Ensures sufficient nutrition despite reduced feed intake	[11]
Environmental Management	Improved ventilation and cooling systems	Lowers ambient temperatures and reduces the thermal load on animals	[63]
Environmental Management	Optimized housing design (reflective roofing, insulation)	Enhances animal comfort by minimizing heat accumulation	[64]
Genetic	Selection for heat tolerance	Breeds animals inherently more resilient to high temperatures while maintaining productive traits	[66]

## Data Availability

No new data were created or analysed in this study. Data sharing is not applicable to this article.
